# Effect of Mold Temperature on the Impact Behavior and Morphology of Injection Molded Foams Based on Polypropylene Polyethylene–Octene Copolymer Blends

**DOI:** 10.3390/polym11050894

**Published:** 2019-05-15

**Authors:** Santiago Muñoz-Pascual, Eduardo Lopez-Gonzalez, Cristina Saiz-Arroyo, Miguel Angel Rodriguez-Perez

**Affiliations:** 1 Cellular Laboratory (CellMat), Universidad de Valladolid, 47011 Valladolid, Spain; eduardol@fmc.uva.es (E.L.-G.); marrod@fmc.uva.es (M.A.R.-P.); 2 CellMat Technologies S.L., Paseo de Belen 9-A (CTTA Building), 47011 Valladolid, Spain; c.saiz@cellmattechnologies.com

**Keywords:** polypropylene, POE, foam, impact strength, elastomer morphology, crystalline morphology, cellular structure

## Abstract

In this work, an isotactic polypropylene (PP) and a polyethylene–octene copolymer (POE) have been blended and injection-molded, obtaining solids and foamed samples with a relative density of 0.76. Different mold temperature and injection temperature were used. The Izod impact strength was measured. For solids, higher mold temperature increased the impact resistance, whereas in foams, the opposite trend was observed. In order to understand the reasons of this behavior, the morphology of the elastomeric phase, the crystalline morphology and the cellular structure have been studied. The presence of the elastomer near the skin in the case of high mold temperature can explain the improvement produced with a high mold temperature in solids. For foams, aspects as the elastomer coarsening in the core of the sample or the presence of a thicker solid skin are the critical parameters that justify the improved behavior of the materials produced with a lower mold temperature.

## 1. Introduction

Polypropylene (PP) is a polymer with relatively low cost and excellent properties such as low density, easy processability, good recyclability, moisture resistance, high thermal stability or excellent chemical and corrosion resistance. These versatile properties make this material one of the most used commodity plastics, and this is the reason why the mechanical performance of PP arouses scientific interest. PP exhibits good stiffness and strength, but its use in certain applications is somehow limited by its impact strength, that it is specially reduced at low temperatures, due to its high degree of crystallinity and its high glass transition temperature [1,2,3]. Consequently, it could be said that there is a gap between its mechanical properties and that of the engineering plastics, limiting its use for certain applications. 

However, this gap can be adequately filled by applying certain approaches such as the modification of the crystalline structure, the addition of fillers, reinforcements, etc. [4]. One option is related to a proper selection of the polymorphism of the crystalline structure in PP (α, β, γ and smectic mesophase can be obtained). It is known that the β-phase of PP outperforms α-PP in toughness [5]. However, unless highly selective nucleating agents are used, PP typically crystallizes in the α-form, this morphology being the one obtained in this study [5]. Another common strategy is to blend PP with other polymers, especially with elastomers [6]. The key to obtain an impact improvement is the contribution of the dispersed elastomeric phase via crazing, shear yielding and cavitation [7,8,9]. A large amount of studies has been carried out using different types of elastomeric phases such as poly(styrene–ethylene–butylene–styrene) (SEBS) [2,10], ethylene–propylene rubber (EPR) [11,12] or ethylene propylene–diene monomer elastomer (EPDM) [1,13]. One of the most promising groups of elastomers is composed by the ethylene/α-olefin copolymers or polyolefin elastomers (POE). The most used are based in α-olefins as octene or butene. Polyethylene–octene copolymers provide properties such as good shear thinning behavior, melt elasticity and melt processability [14]. 

Microstructure in polymer blends is very complex and crucial in the mechanical properties and especially in impact response [12]. Wu [15,16] proposed that the interparticle distance (or matrix ligament thickness) is a key factor that dominates the brittle–tough transition of polymer blends. If the interparticle distance (ID) is smaller than a critical value (IDc), the blend is tough, otherwise the blend is brittle. For PP, a critical value of 0.42 μm was reported [17]. Due to this, it is necessary to apply a proper processing approach in order to reach the minimum interparticle distance between the elastomer particles. The minimum distance between particles is inversely proportional to the diameter of the elastomeric phase, following the theoretical relation proposed by Wu [18] and modified by Liu [19,20].

(1) $$ID=d\left[{\left(\frac{\pi }{6\phi }\right)}^{1/3}-1\right]$$

(2) $$ID=d\left[{\left(\frac{\pi }{6\phi }\right)}^{1/3}\exp\left(1.5ln^{2}\sigma \right)-\exp\left(0.5ln^{2}\sigma \right)\right]$$

where ID is the interparticle distance, d is the diameter of the particle, ϕ is the rubber volume fraction and σ is obtained from the distribution of particle sizes using Equation (3):

(3) $$ln\sigma =\sqrt{\frac{\sum_{i=1}^{N}n_{i}{\left(lnd_{i}-lnd\right)}^{2}}{\sum_{i=1}^{N}n_{i}}}$$

where $n_{i}$ is the number of particles with diameter $d_{i}$ and N is the total number of particles (σ is equal to one for a monodisperse distribution and it is higher than one for a polydisperse distribution). 

An example that proves the importance of the distance between particles in the impact properties of PP/POE binary system could be found in Premphet study [21]. 

Processing can be the determinant in the obtained microstructure. In extruders and blend mixers, some general correlations between the rheological properties of the polymer blends and the particle size of the dispersed phase were deduced by Wu and Tokita [18,22,23]. Moreover, the coalescence and relaxation of polymer blends in the molten state has a significant influence on the final morphology [24,25,26,27]. Injection molding is the most extended process in the plastic industry, due to its high versatility (a wide range of part geometries are possible), short cycle time and good surface quality. PP/POE blends have been studied by several authors, such as Li, Liu and Ying [28,29,30], describing the elastomer phase morphology along the profile of the injecting molded solid material. These previous results for solids will be used in the discussion section of this paper.

On a separate issue, foaming can improve properties of polymers such as thermal insulation or acoustic properties [31,32] whereas the density is reduced, creating material savings and being key in weight-limited applications. Moreover, foams can be used in a great variety of sectors such as packaging, construction, automotive, and comfort applications [33]. In the case of foamed parts based on PP, it can be said that the impact resistance is poorer than that of solid PP. A dramatic reduction in impact strength is typically achieved in PP based foams even at high relative densities. There is a strong dependence of the properties of cellular materials (like elastic modulus, strength or thermal conductivity) with density. Any property can be estimated using the scaling (or power) law models [34]. These empirical relationships relate a certain property of the cellular material ($X_{foam}$) with the same property of the corresponding solid polymer ($X_{solid}$) and its relative density (ratio between the density of the foam ($\rho_{foam}$) and the density of the corresponding density ($\rho_{solid}$)) (Equation (4)):

(4) $$X_{foam}=C\cdot X_{solid}\cdot {\left(\frac{\rho_{foam}}{\rho_{solid}}\right)}^{n}$$

Typically, C exhibits values close to one and n reaches values between n = 1 and n = 2, depending on the cellular structure and the considered property (for example, elastic modulus). A high value of n implies a significant lowering of the foam property when the density is reduced, which means that if reaching a high value of this property is critical for the final use of the item, the foaming of this particular item would not be suitable. 

Throne [35] studied the mechanisms underlying the impact response of structural foams (foams with well-defined solid skins) based on polyphenylene oxide (PPO) and polystyrene (PS), proving the existence of a ductile–brittle transition appearing even at high relative densities. This means that even the most ductile polymers can fail in a brittle manner if they are foamed and subjected to high strain rates. The proposed scale law for the impact energy during an impact test was (Equation (5)):

(5) $$I_{foam}=I_{solid}{\left(\frac{\rho_{foam}}{\rho_{solid}}\right)}^{m}{\left(\frac{h_{foam}}{h_{solid}}\right)}^{n}$$

where $I_{foam}$ and $I_{solid}$ are the absorbed energies during the impact for the foamed and the non-foamed sample respectively, $h_{foam}$ and $h_{solid}$ are the thickness of the foam and solid samples respectively, $\rho_{foam}$ is the density of the foam and $\rho_{solid}$ the density of the solid specimen.

The first approximation of Throne for thermoplastics was m = 4 and n = 2. On the other hand, Tejeda et al. [36] denoted that most of the polymers exhibit a performance following trends with values of m and n close to 2 and 3 respectively, but for PP, the adequate values were m = 3 and n = 1. The high values reached by m in all cases reflect the magnitude of the ductile–brittle transition problem, that limits the application of PP foams in sectors like automotive or aeronautics in which the plastic parts must fulfill stringent mechanical requirements at high strain rates. Foaming injection molding is becoming more and more used in the industry due to several advantages, such as the possibility of producing complex parts and the possibility of producing structural foams (i.e., foams with solid skins in the surface), which is known to allow the improvement of the mechanical performance in comparison with conventional foams [35,36,37]. This specific advantage added to the benefits already mentioned for solid plastics make this technology a good approach to produce plastic parts with complex geometries for industrial applications. Taking into account that the use of POE elastomers has been successful in improving the impact behavior of PP, it is logical to apply the same concept to PP based foams. However, this approach has not been studied in detail so far. The only work, written by Gong et al. [38], studied the impact resistance of foamed and solid materials based on PP/POE blends, with POE contents ranging from 0 wt.% to 20 wt.%, and low (−80 °C) and high (20 °C) impact temperatures. The materials were produced using similar mold and nozzle temperature. They showed qualitatively the elastomer morphology, but they did not study in detail important parameters such as interparticular distance or deformation of the dispersed phase. In addition, they did not analyze the crystalline morphology of foams or solids.

Taking the previous ideas in mind, the main objective of this research is to gain new knowledge on the complex impact response of foamed PP/POE blends, with the final target of creating low density foams with show a ductile impact response with high impact strengths. As it has been explained above, the morphology and impact properties of solid PP/POE blends has been the subject of several studies. However, as far as the authors know, the mechanical behavior and the complex morphology of foams based on PP/POE blends have not been previously studied in detail. The detailed analysis of the morphology of the samples will be determined in order to explain the variations in mechanical performance. In addition, the effect of processing conditions such as the nozzle temperature and the mold temperature has not been yet considered in the literature. 

## 2. Materials and Methods 

### 2.1. Materials

Isotactic polypropylene (iPP) ISPLEN PP080G2M was purchased from Repsol (Madrid, Spain) with a melt flow index of 20 g/10 min at 230 °C and a density of 0.905 g/cm^3^, being a low viscosity degree suitable for injection molding process. Ethylene–octene copolymer (POE) Engage 8137, with melt flow index (MFI) of 13 g/10 min at 190 °C and a density of 0.864 g/cm^3^ was purchased from Dow, Midland, USA. This degree was chosen by its easy processability. The used blowing agent was Hydrocerol BIH 40 provided by Clariant. An endothermic blowing agent, it is based on sodium bicarbonate and citric acid. Antioxidants Irgafos 168 (from Ciba, Basel, Switzerland) in a proportion of 0.08 wt.% and Irganox 1010 (from Ciba, Basel, Switzerland) in a proportion of 0.02 wt.% were added to the formulations to prevent thermal oxidation of the polymer during blending.

### 2.2. Experimental Methods

#### 2.2.1. Sample Production

PP and POE pellets and the aforementioned antioxidants were mixed and blended in a twin-screw extruder (Collin Teach Line model ZK 25T SCD 15, Maitenbeth, Germany) with a temperature profile of 145, 150, 155, 160, and 165 °C from the hopper to the die and a screw speed of 200 rpm. The election of these conditions was determined by the need to increase the shear rate in order to obtain a low particle size of the elastomeric phase. The POE content was 20 wt.%. 

Solid and foamed materials were produced using a microinjection-molding machine (BabyPlast 6/10P) varying two processing parameters, the melt temperature and the mold temperature. The injection temperature from the rear to the nozzle and the mold temperature are summarized in Table 1. The mold temperature was varied between 30 °C and 50 °C. The used nomenclature reflects the temperature of the nozzle and the mold temperature level: Low (L) or high (H).

**Figure 1:**
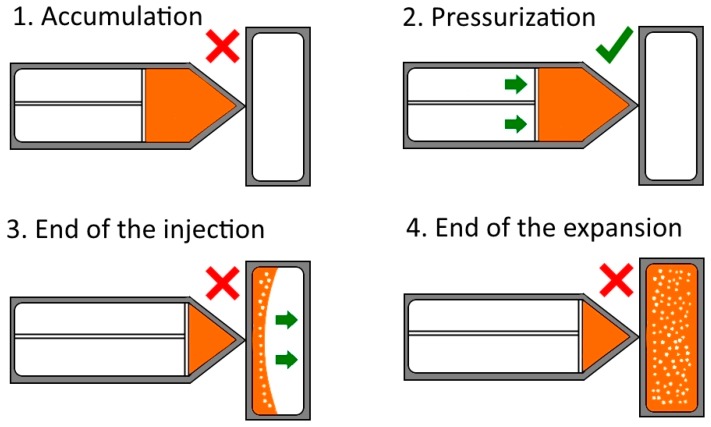
Schematic stages of the low-pressure injection molding process. The green tick (**red cross**) indicates the aperture (**closure**) of the valve between the nozzle and the mold.

The foamed samples were produced using the so-called low-pressure foaming injection molding process [37]. A scheme of the process can be observed in Figure 1, detailing its different stages. 

In this process, the polymer containing the gas phase that comes from the thermal decomposition of the blowing agent was introduced into the mold cavity in short injection shots. The injection pressure in our case was 11 MPa. In low-pressure injection molding, the injection shot amounts to only 65–80% of the mold cavity. Then, the polymer expands, filling the mold and the cellular structure is formed. Then, the density reduction depends only on the injection shot. The amount of released gas strongly influences the cellular structure. As the difference of gas released was similar for all the nozzle temperatures (checked by Thermogravimetric analysis (TGA) experiments), a fixed percentage of blowing agent (2 wt.%) was added for all the samples and all foaming conditions. The relative density of the obtained foamed samples was 0.76, meaning a density reduction of 24%. The dimensions of the solid and foamed materials were 81 × 14.50 × 4 mm.

#### 2.2.2. Mechanical Testing

The notched Izod impact strength of the specimens was tested using a Frank 53566 Izod pendulum according to the UNE-EN ISO 180/A standard. The experiments were carried out at 23 ± 2 °C and 50% ± 10% relative humidity according the ISO 291 standard, and the average value was obtained from testing over seven specimens for each material. The injection molded samples were machined to a size of 74 × 10 × 4 mm removing the lateral skin of the samples. The v-notch depth was 2 mm.

#### 2.2.3. Structural Characterization

To characterize the cellular structure of the foamed samples, the materials were cooled down in liquid nitrogen and then fractured. A thinner layer of gold was sputtered on the fractured surface to make it conductive. The micrographs were taken using a Hitachi FlexSEM 1000 scanning electron microscope (SEM, Hitachi, Chiyoda, Japan). The average cell diameter was determined using an image-processing tool based on ImageJ software [39]. Relevant statistical parameters such as the standard deviation (SD) of the cellular structure distribution were calculated according to Equation (6).

(6) $$SD=\sqrt{\overset{n}{\underset{i=1}{\sum }}\frac{{\left(\phi_{i}-\phi \right)}^{2}}{n}}$$

where n is the number of counted cells, $\phi_{i}$ is the cell diameter of cell i and ϕ is the average diameter of the cells. This parameter accounts for the width of the cell size distribution. The skin thickness in the foamed materials was measured in several points along the profile of the sample with ImageJ and the average value was obtained.

To evaluate the elastomer phase dispersion, the specimens were cooled down in liquid nitrogen and then fractured. The resulting surface was etched with xylene at room temperature during 15 h to remove the elastomer phase from the iPP matrix. Later, gold was sputtered, and SEM images were taken for the following distance from the skin: 50, 100, 200, 500, 1000 and 2000 μm. The distribution of the distances to the nearest neighbor were obtained using ImageJ software. It is important to remark that this distance was the edge to edge distance and was calculated measuring the distance for each particle to all of them and obtaining the minimum value. 

The deformation of the particles was calculated following the equation (Equation (7)):

(7) $$D=\frac{L-S}{L+S}$$

where L and S are respectively the long and the short axis. 

The average of the distributions of interparticle distance and deformation was calculated.

To evaluate the crystalline morphology, the specimens were cooled down in liquid nitrogen and then fractured. The resulting surface was etched with a dissolution of 7 wt.% of potassium permanganate in sulfuric acid for one hour. Then, the samples were cleaned in water and hydrogen peroxide, using ultrasound technique. Finally, gold was sputtered, and SEM images were taken. From these images, the crystalline morphology observed in the samples was qualitatively analyzed.

## 3. Results

### 3.1. Mechanical Properties

**Figure 2:**
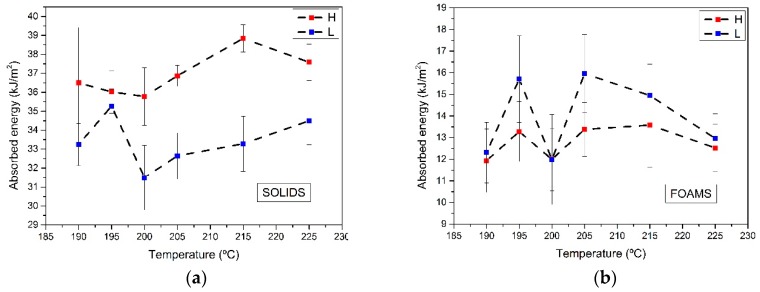
Absorbed energy in the Izod impact test for (**a**) solid and (**b**) foamed samples.

The obtained values of the notched Izod impact test for the solid and foamed samples are summarized in Figure 2.

As expected, the absorbed energy by foamed samples was less than half the values of the solid materials. The n-exponent in Equation (4) was near (but higher) than n = 3 (the average value for all samples analyzed was 3.33). This value of the exponent indicates that a reduction of 24% in weight produced a decrease in impact resistance of 60%. Despite this, the fracture was ductile for all the analyzed materials, i.e., it can be said that for the materials under study the ductile–brittle transition was not observed. There is no a clear trend of the impact strength of both solid and foams as a function of the nozzle temperature. All the results are within the experimental uncertainty. However, there is a clear effect of mold temperature. For all the solid materials, the absorbed energy for high mold temperature samples is over the value obtained for the low temperature mold samples. In fact, the average of the absorbed energy for the high mold temperature solid samples is about 11% higher than that of the low temperature solid materials. In foams, the trend is the opposite, the materials produced with a lower mold temperature present a higher absorbed energy. In particular, 9% higher. 

In order to understand this surprising behavior, a detailed characterization of the elastomeric phase morphology, crystalline morphology and cellular structure of the samples was needed. The samples produced with a nozzle temperature of 215 °C were selected for this characterization. The reason to select this temperature was that the difference of the impact resistance reached a maximum at that temperature in the solid samples.

### 3.2. Elastomer Phase Morphology

**Figure 3:**
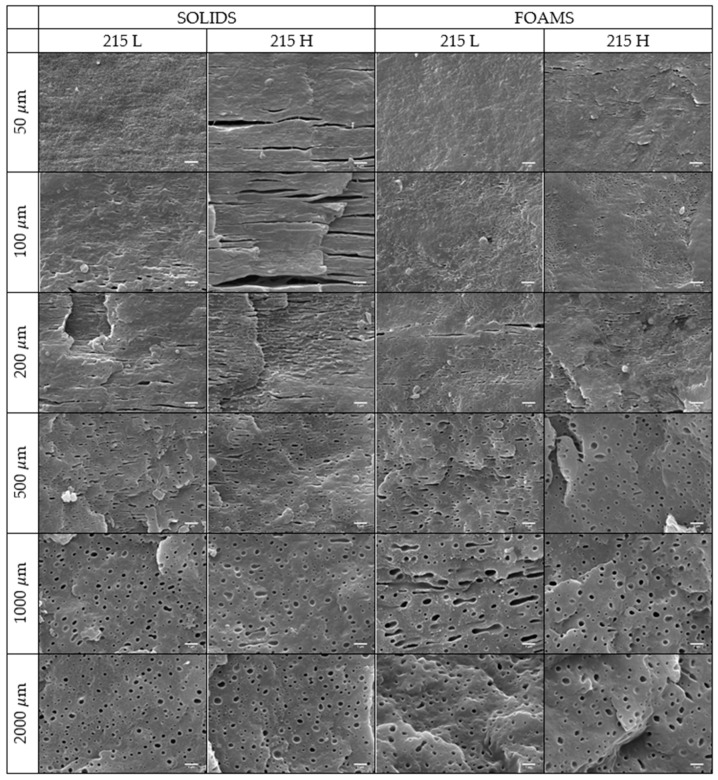
SEM images of xylene etched samples for different distances from the skin of the injection molded sample. In the core of the foam, images were taken in the interior of the walls. The scale bar represents 1 μm.

Figure 3 shows representative SEM micrographs of both the solid and foamed samples produced with different mold temperature in the Machine direction (MD) plane. This direction was selected because in previous papers [28,29,30] the hierarchical structure of the materials was better observed in this plane.

It can be observed that there is a heterogeneous distribution of the elastomeric phase in all the analyzed materials. Near the skin, until 100 μm, no particles are detected except in the 215 H solid sample, in which it can be appreciated the existence of extremely elongated droplets. As the distance from the skin is raised, elastomer particles appear in all the samples, with higher elongation in the case of the solids. In the core (1000–2000 μm), the particles are more spherical, with a large size in the case of foams.

**Figure 4:**
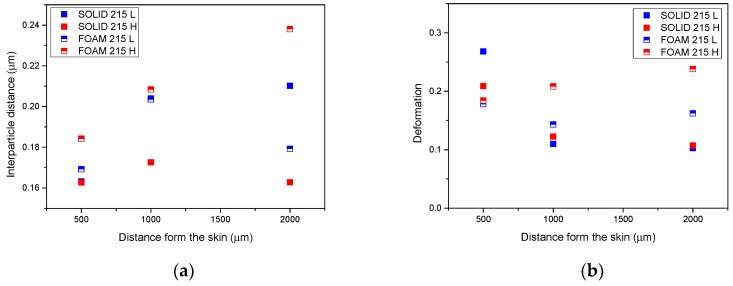
(**a**) Interparticle distance and (**b**) deformation for 215 low (L) and 215 high (H) foamed and solid samples.

These observations are supported by the distribution analysis of the interparticle distance (ID) and deformation (D) of the particles in the core of the samples (Figure 4). In the interparticle distance graph (Figure 4a), the increment of this parameter is appreciated as the distance from the skin is increased, which is in agreement with the observed increment of particle diameter in the core in Figure 3. Moreover, it can be observed at 2000 μm from skin that whereas for the solid samples the interparticle distance is higher for the 215 L sample, for the foamed specimens, the results are the opposite and the highest value is reached for the 215 H sample.

In the case of the deformation (Figure 4b), the reduction of the deformation for solids and the constant value in foams when we approach the core of the samples can be observed. In addition, the deformation of foamed samples is higher which could be due to coalescence of several particles.

### 3.3. Crystalline Morphology

**Figure 5:**
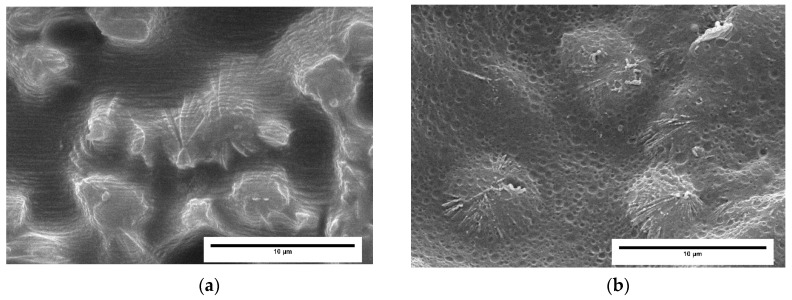
SEM images of a permanganic etched solid sample (215 L) at (**a**) 100 μm and (**b**) 2000 μm from the skin. The scale bar represents 10 μm.

The reference 215 L solid was etched with a permanganic solution to observe the crystalline structure present in the produced samples. As it can be seen in Figure 5, there are clear differences between the skin and the core of solids.

**Figure 6:**
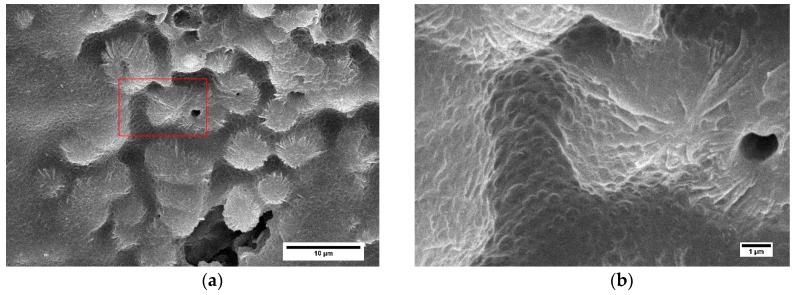
SEM images of a permanganic etched foamed sample (215 L) at 2000 μm from the skin, in the wall of a cell. The image (**b**) is a zoom of the selected zone of the micrograph to appreciate the elastomeric phase (**a**).

Near the skin (Figure 5a), the flow has orientated the morphology and as a result the shish-kebab morphology appears. This superstructure is caused by high shear rates during crystallization. Remarkable enhancement in mechanical properties (impact resistance and tensile strength) are typically observed in neat PP with this structure [40]. The shish-kebab content is higher when the mold walls are cooler. It occurs because, in a situation with a higher mold temperature, the slow cooling of the melt in the interior part allows oriented chains to relax before crystallization [40]. Then, in our case this layer was thicker for lower temperature mold samples (higher cooling rate), and it corresponds with the area where no elastomer was detected. High mold temperature condition reduced the shear in the injecting molded samples and allowed the inclusion of elastomer in layers nearer the skin. On the other hand, in the core it is possible to observe the presence of the elastomer particles into the spherulites and between them. This structure is favored by slow cooling rates, which in our study would correspond to the solid samples produced using a higher temperature in the mold and to the foamed samples. If fact, this structure can be clearly appreciated in the cell walls of the foamed materials (Figure 6). In this case, the perfect inclusion of the elastomer particles in the spherulites can be inferred. 

### 3.4. Cellular Structure

**Figure 7:**
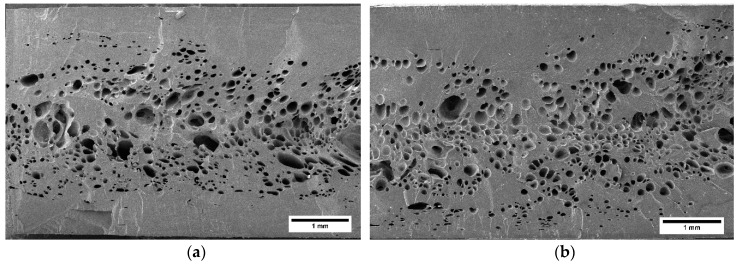
TD plane of (**a**) 215 L and (**b**) 215 H foams. The scale bar represents 1 mm.

Figure 7 shows the cellular structure of the foams in the TD (Transverse direction) plane. They present the typical cellular structure for injection molded foams characterized by the presence of a solid skin and a foamed core.

The analysis of the solids skin thickness revealed that the 215 L material presented with a clearly thicker skin. For this material, the skin represented 38% of the total thickness, clearly higher than the 24% of the 215 H sample.

Regarding the internal cellular structure, both materials present similar cell sizes and standard deviation of the cellular structure as can be observed in Table 2. Cell sizes were in the range of 100–125 microns and standard deviation of the cellular structure distribution was between 40 and 60 microns. This cellular structure distribution is typical of the low-pressure injection molding process, which is characterized by a low uniformity [41]. 

## 4. Discussion

**Figure 8:**
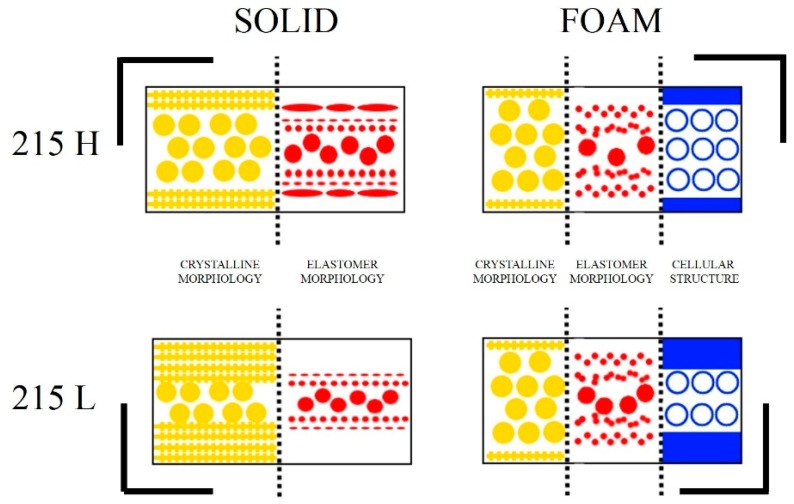
Schematic model of the elastomer and crystalline morphology in the case of solids, adding the cellular structure in the case of the foamed samples.

It is well known that solid PP/POE systems are complex materials. Its morphology should be analyzed at least at two different levels: Elastomeric morphology and crystalline morphology. In addition to this, for foamed materials we need to consider the morphology of the cellular structure and the presence of a non-foamed solid skin. In order to facilitate the understanding of the results obtained, a schematic model with the observed structures is presented in Figure 8.

This figure shows the following key results:

- The skin was free of elastomeric particles and the crystalline morphology was characterized by a shish-kebab structure. Spherulite morphology was predominant in core zones, allowing the inclusion of elastomer particles.
- The solid produced with a high mold temperature exhibited a zone of elongated elastomeric particles from 50 μm to 500 μm.
- On the other hand, in the solid produced with a low mold temperature, the elongated particles were only observed for distances from the skin greater than 200 μm.
- In foams, particles were less elongated in the layers nearer the mold walls and the transition from skin to the core was less abrupt than for solids. Moreover, elongated particles were observed in more internal positions that seemed to be produced by coalescence of several particles.
- Spherical particles were present in the core of solids and foams. The elastomer particles are more separated in 215 L than 215 H solids. In foams, the opposite result was observed.
- The cellular structure analysis determined that the cell size was very similar for the two conditions used, but the skin was thicker in the case of the low temperature mold foam (Table 2) (about 60% thicker).

In solids, this hierarchy structure, composed by layers, has been well studied [28,29,30]. It is well known that morphology of polymer blends is determined by the flow history and the properties of the components [42]. It is clear that the flow fields can deform the elastomer particles in the blend, and the final geometry of them will be determined by two magnitudes: The capillary number Ca and the viscosity ratio p [42] (Equations (8) and (9)):

(8) $$Ca=\frac{\eta_{m}R\dot{\gamma }}{\Gamma }$$

(9) $$p=\frac{\eta_{d}}{\eta_{m}}$$

where $\eta_{m}$ and $\eta_{d}$ are the viscosity of the matrix and the dispersed phase, R is the initial radius of the dispersed phase droplet, $\dot{\gamma }$ is the shear rate and Γ is the interfacial tension. The influence of these relations can be understood in the following way. Ca represents the resistance to deformation of a particle, being easier to deform with the increment of the particle radius, the increment of the viscosity matrix and higher values of shear rate. On the other hand, the interfacial tension increment reduces the deformability of the particle. The viscosity ratio is crucial. If value p is high, the matrix viscosity is too low to change the shape of a dispersed phase with high viscosity [28,42]. When Ca is above a critical value (dependent on the value of p), the droplet attains a steady shape and orientation, whereas above the critical capillary number the droplet eventually breaks up, by the apparition of a capilarity-wave instability. These effects have been summarized by Moldenaers and Tucker in a review [42].

The influence of this crystalline structure in PP/POE blends were studied by Geng [43]. These experiments were carried out using a technique called dynamic packing injection molding (DPIM), which imposed oscillatory shear on the gradually cooled melt during the packing solidification stage. For low POE content, the DPIM samples were tougher than traditional injection molding due to shish-kebab crystal structure developed due to the high orientation. When a higher content of POE was added, the effect over these samples was negligible due to the inability of the elastomer to flow plastically in the highly oriented matrix. To summarize, the orientation of matrix and elastomer were crucial in PP toughening. Then, for our case, it can be inferred that a higher shish-kebab structure caused an ineffective toughening of the PP when POE was added. Hu [44] and Du [45] also experimented with crystal morphology, orientation and elastomers. Despite of using β crystalline morphology in their studies, the conclusions were that the crystal must be well developed in order to obtain a proper elastomer reinforcement in PP.

Taking into account the previous discussion, it is logical to conclude that the shish-kebab structure layer is thicker in the low mold temperature sample: Increasing the cooling rate causes a faster crystallization under higher shear conditions. A narrower shish-kebab layer can explain why elongated elastomer particles are present in skin layer for high mold temperature solids and not in the low ones. The presence of this elastomer in the high temperature case could improve the impact response observed in these samples. Moreover, as the previous publications reported, the elastomer particles in high shear layers perform poorly, as could be occurring in our low mold temperature solids. In addition, the interparticle distance in the core, despite of being below the critical value, is higher in the case of the 215 L sample, which is typically associated with a lower toughening.

To analyze the behavior of the foams, it is necessary to analyze the characteristics of the polymer–gas blend when it is introduced in the mold. Quin et al. studied the injection of foams focusing in the viscosity of the blend, reaching the following equation (Equation (10)) [46]:

(10) $$\eta_{r}=\frac{\eta_{s}}{\eta_{m}}={\left(1-\phi_{g}\right)}^{a}$$

where $\eta_{r}$ was the relative viscosity, $\eta_{s}$ the viscosity of the gas/polymer suspension, and $\eta_{m}$ the viscosity of the polymer, $\phi_{g}$ is the gas volume fraction and a is a parameter that is between one and two. The conclusion was that the viscosity of the blend was lower when gas was added. Due to this, a reduced viscosity of the polymer blends is expected when producing the foams. Therefore, considering Equation (8), we can conclude that the elastomer particles should be less deformed and, if they were, the shear rate was not enough to break-up them. As it can be seen in Figure 3, the deformation in the shear band was not so clear, and even so, it is possible to observe particles that have been deformed but they have not broken up. So, shear associated effects have no incidence in foams (at least, not as much as in solids). In addition, it can be appreciated in Figure 4 that the deformation of the particles in foamed materials did not suffer a reduction as the one observed in the solid materials.

Therefore, the difference in absorbed energy was lower than in the solid case, but the materials produced with a low temperature mold had an impact resistance above those produced with the high temperature mold. Two phenomena could be the responsible for this result, the first of them is evident: The existence of a thicker skin layer when the mold walls were cold is typically associated to an improved mechanical performance due to an optimized distribution of the mass. The second seems to be associated to a higher coarsening produced in high-temperature samples, due to the lower cooling rate (in foam, this problem was worsened in comparison with the solids, due to the low thermal conductivity of the materials). Figure 4 shows an increment in the interparticle distance of 30% for the foamed samples produced using the high mold temperature, and this should result in a reduction of the impact strength. 

## 5. Conclusions

The impact strength and the morphology of foamed materials produced from PP/POE blends have been studied. The foams were produced by using the low-pressure injection molding technology, varying the nozzle temperature and the mold temperature. A 24% density reduction (in comparison with solids materials) was obtained and this density reduction promoted, on average, a 60% reduction of the impact resistance. However, it is important to remark that all the foamed materials presented a ductile behavior, avoiding the typical ductile to brittle transition when PP is foamed. 

Interestingly, the effect of the mold temperature was completely different for solid and foamed materials. For solids, a higher mold temperature promoted a better impact resistance, however for the foamed materials the opposite behavior was observed. The improvement in solids for high mold temperatures was due to a lower shear and slow crystallization, that reduced the presence of a shish-kebab structure in these samples and allowed the presence of an elastomer phase nearer the skin of the materials. Also, a lower interparticle distance in the core of these samples was observed. On the other hand, for the foams the low shear rate caused by the presence of gas in the blend suppressed the crystallization effects near the surface of the sample. Due to this, the behavior was controlled by the presence of a solid skin (thicker when the mold temperature was reduced) and the coarsening of the elastomer in the core (increased by the slow cooling).

The results presented in this paper allow obtaining a better understanding of the complex impact behavior of these bends when they are foamed by injection molding and show promising results in the ultimate goal of creating formulations allowing the production of low density foamed parts that are ductile and have a high impact strength. 

## Figures and Tables

**Table 1:** Different processing conditions for each reference.

Sample Name	Rear (°C)	Middle (°C)	Nozzle (°C)	Mold (°C)
190 L	170	180	190	30
190 H	170	180	190	50
195 L	175	185	195	30
195 H	175	185	195	50
200 L	180	190	200	30
200 H	180	190	200	50
205 L	185	195	205	30
205 H	185	195	205	50
215 L	195	205	215	30
215 H	195	205	215	50
225 L	205	215	225	30
225 H	205	215	225	50

**Table 2:** Cell size, standard deviation and skin thickness of 215 L and 215 H foamed samples.

Sample	$\phi_{3D}\;(\mathrm{mm})$	SD	Skin (%)
215 L	107.96	46.63	38.20
215 H	126.13	60.01	24.14
